# Gastric Emptying Is Not Delayed and Does Not Correlate With Attenuated Postprandial Blood Flow Increase in Medicated Patients With Early Parkinson's Disease

**DOI:** 10.3389/fneur.2022.828069

**Published:** 2022-02-24

**Authors:** Thomas Hartwig Siebner, Stefan Fuglsang, Christopher Fugl Madelung, Annemette Løkkegaard, Flemming Bendtsen, Jens Dahlgaard Hove, Morten Damgaard, Jan Lysgård Madsen, Hartwig Roman Siebner

**Affiliations:** ^1^Danish Research Centre for Magnetic Resonance, Centre for Functional and Diagnostic Imaging and Research, Copenhagen University Hospital—Amager and Hvidovre, Copenhagen, Denmark; ^2^Department of Cardiology, Copenhagen University Hospital—Amager and Hvidovre, Copenhagen, Denmark; ^3^Department of Clinical Physiology and Nuclear Medicine, Centre for Functional Imaging and Research, Copenhagen University Hospital—Amager and Hvidovre, Copenhagen, Denmark; ^4^Department of Neurology, Copenhagen University Hospital—Bispebjerg and Frederiksberg, Copenhagen, Denmark; ^5^Department of Clinical Medicine, University of Copenhagen, Copenhagen, Denmark; ^6^Gastrounit, Medical Division, Copenhagen University Hospital—Amager and Hvidovre, Copenhagen, Denmark

**Keywords:** Parkinson's disease, non-motor symptoms, gastric emptying, scintigraphy, gastrointestinal dysfunction, postprandial blood flow

## Abstract

**Background:**

We have recently used phase-contrast magnetic resonance imaging (PC-MRI) to demonstrate an attenuated postprandial blood flow response in the superior mesenteric artery (SMA) in 23 medicated patients with Parkinson's disease (PD) compared to 23 age- and sex-matched healthy controls.

**Objective:**

To investigate in a sub-sample of the original cohort whether the observed blood flow response in SMA after oral food intake is related to a delay in gastric emptying.

**Methods:**

We studied 15 patients with PD in an “ON-medication” state with a mean disease duration of 3.9 ± 2.2 years and 15 healthy age- and sex-matched individuals. Participants underwent dynamic gastric scintigraphy 0, 30, 60, 120, 180 and 240 minutes after the intake of a standardized radiolabeled test meal. Gastric emptying was compared between groups. 14 of the 15 PD patients and 12 of the 15 healthy control subjects had previously undergone serial postprandial PC-MRI measurements. In these individuals, we tested for a relationship between gastric emptying and postprandial blood flow response in the SMA.

**Results:**

The dynamics of gastric emptying did not differ between groups (*p* = 0.68). There was substantial inter-subject variability of gastric emptying in PD patients and healthy participants. Only a single PD patient had delayed gastric emptying. In those participants who had undergone PC-MRI, postprandial increase in SMA blood flow was attenuated in PD compared to healthy controls as reported previously (*p* = 0.006). Gastric emptying did not correlate with the timing and amplitude of postprandial blood flow increase in SMA.

**Conclusion:**

Our preliminary results, obtained in a small group of early-stage PD patients who continued their usual dopamine replacement therapy, suggest that variations in gastric emptying after solid meal intake is within the normal range in the majority of cases. There is also no evidence for a tight relationship between the attenuated postprandial blood flow response in the SMA and normal variations in gastric emptying.

## Introduction

Gastrointestinal dysfunction is one of the main disabling non-motor symptoms in Parkinson's disease (PD) and occurs at all stages of PD (1–4). Gastrointestinal complaints include upper gastrointestinal symptoms such as increased salivation and dysphagia (5–8). However, symptoms that are attributable to delayed gastric emptying, such as early satiety or abdominal bloating (9, 10), are not more frequently present in PD patients than healthy subjects (5, 6, 8).

Gastric emptying has been studied in PD patients to objectify functional impairment in PD. Dynamic scintigraphy of gastric emptying after ingestion of a radioactively labeled solid meal is considered to be the gold standard to estimate gastric emptying time (GET) (11, 12). Previous studies that have used dynamic scintigraphy after oral intake of a solid or liquid meal have revealed conflicting results, when comparing GET in PD patients and healthy individuals (Table 1). A direct comparison of these studies is difficult because they differ substantially with respect to the patients' medication, disease duration and severity, and age span of healthy control participants. Most scintigraphy studies have found a substantial inter-individual variation in GET in both PD patients and healthy participants with largely overlapping ranges in the two groups.

**Table 1:** Demographic and scintigraphic gastric emptying data from studies of patients with Parkinson's disease (PD) in comparison with healthy controls (HC) in the last 25 years.

**Study** **(meal type)**	**Subgroups** **(*n*)**	**Mean age (years ±SD)**	**Mean gastric half-emptying time (*T*_a/b_) (minutes ±SD)**	**Range of Hoehn and Yahr stage**	**Mean disease duration (years ±SD)**	**ON- or OFF- medication state**
**Djaldetti et. al**. **(****13****)** (Solid meal)	HC (22)	45 ± 3.5	56 ± 5			
	PD (15)	62 ± 9	221 ± 202	2-4	8.8 ± 4.6	ON with fluctuating symptoms (presence of “wearing-off and “delayed-on” phenomena)
	PD (15)	64.8 ± 9.9	85 ± 31	1-4	4.0 ± 3.3	ON without response fluctuations
**Hardoff et al**. **(****14****)** (Solid meal)	HC (22)	61.9 ± 6.1	43.4 ± 10.8			
	**Subdivided by disease severity**					
	PD (29)	61.5 ± 5.9	63.4 ± 28.8	1–2	3.6 ± 3.6	8 ON of which 2 with response fluctuations, 21 OFF (untreated)
	PD (22)	65.1 ± 5.6	54.7 ± 25.5	2.5-3	7.1 ± 5.3	15 ON of which 11 with response fluctuations [presence of “wearing-off”, “delayed on”, “no on”, or “on/off” phenomena, 7 OFF (untreated)]
	**Subdivided by treatment status**					
	PD (28)	62.8 ± 5.6	59.5 ± 30.6	-	-	OFF (untreated)
	PD (13)	63.6 ± 6.6	49.3 ± 16.2	-	-	ON with fluctuating symptoms
	PD (10)		73.6 ± 25.8	-	-	ON without response fluctuations
**Krygowska-Wajs et al**. **(****15****)** (Solid meal)	HC (15)	59.5 ± 9.7	38.4 ± 7.3			
	Familial PD^*a^ (10)	59.0 ± 8.2	58 ± 25	1–3	8.4 ± 5.2	ON
	Sporadic PD (35)	60.5 ± 9.9	46 ± 25	2–4	7.1 ± 4.3	ON
**Gjerløff et al**. **(****16****)** (Solid meal)	HC (12)	62 ± 8	75 ± 22			
	PD (12)	64 ± 9	56 ± 22	1–3	5.3	OFF (withdrawn from anti-parkinsonian medication for 12 h)
**Trahair et al**. **(****17****)** (Liquid meal)	HC (21)	64.8 ± 1.8^*b^	100 ± 29			
	PD (21)	64.2 ± 1.6^*b^	106 ± 42	1–2.5	6.3 ± 0.9^*b^	3 ON, 18 OFF (withdrawn from the morning dose of anti-parkinsonian medication)
**Knudsen et al**. **(****18****)** (Solid meal)	HC (17)	65.4 ± 6.2^*c^	48.2 ± 16			
	PD (22)	64.7 ± 7.1^*c^	50.6 ± 11	1–3^*c^	4.4 ± 4^*c^	ON

*Studies not including a control group or studies recruiting highly selected PD patients were excluded. n, Number of participants; SD, Standard deviation*.

*a *Presence of at least 2 affected individuals within 2–3 consecutive generations in a family*.

*b *Data are presented as mean ± SE*.

*c *Demographic data based on 32 PD patients and 26 healthy control subjects, respectively*.

*Missing data from Gjerløff et al. and Trahair et al. were obtained from the corresponding authors by Knudsen et al. (11) and are marked in red*.

Impaired gastric emptying has been attributed to alterations of gastric motility (1, 19). Scintigraphic assessment of antral contraction has not shown any significant difference in frequency or amplitude between patients with mild & moderate PD and healthy subjects (14). While real-time MRI of gastric motility has revealed a reduction in the amplitude of gastric peristaltic contractions in patients with PD compared to healthy controls, overall gastric motility does not seem to be reduced in PD (20). Another MRI study showed that PD patients with either early satiety or epigastric pain have decreased gastric motility and delayed gastric emptying compared to patients without these symptoms, but the study design did not include a comparison with healthy subjects (21). Gastric contraction frequency has also been assessed using an electromagnetic capsule system, showing no alterations in PD patients compared to younger healthy subjects (22). Taken together, these dynamic studies of gastric motility have failed to provide consistent evidence that gastric motility is impaired in PD.

Using phase-contrast magnetic resonance imaging (PC-MRI), we recently introduced a novel method to study postprandial blood flow regulation in the superior mesenteric artery (SMA) in PD. We found that the increase in SMA blood flow in response to oral intake of a standardized liquid test meal was attenuated in patients with PD compared to healthy individuals without affecting the timing of the SMA response (23). Since the attenuation of the postprandial blood flow increase in the SMA varied substantially from patient to patient, variations in gastric emptying may account for this interindividual variability. Here, we tested this hypothesis using dynamic gastric scintigraphy. The aims of this study were twofold. First, we wished to investigate whether gastric emptying is delayed in PD, and if so, how many patients were affected. Secondly, we wished to clarify whether the attenuation of the postprandial SMA response is associated with prolonged gastric emptying.

## Methods

### Participants

15 patients with PD and 15 healthy age- and sex-matched individuals underwent scintigraphic measurements of gastric emptying after ingestion of a standardized radiolabeled test meal. All participants gave their written informed consent before their participation. The study was approved by the Regional Committee on Health Research Ethics of the Capital Region of Denmark (H-18054923). 14 PD patients and 12 control subjects had previously participated in an experiment, during which postprandial changes in SMA blood flow in response to a standardized liquid test meal intake had been measured with dynamic PC-MRI. These results were recently reported in another publication using a larger cohort of 23 patients with PD and 23 age- and sex-matched healthy control participants (23). We also included one PD patient and three healthy control subjects who had only agreed to participate in scintigraphic assessment of gastric emptying. The recruitment procedures and exclusion criteria have previously been described in detail by Siebner et al. (23). Notably, PD patients and healthy control participants were included regardless of the presence or degree of gastrointestinal symptoms. Patients had to be older than 50 years and be diagnosed with PD by a movement disorder specialist according to the Movement Disorder Society Clinical Diagnostic Criteria for Parkinson's Disease (24).

The demographic and clinical data are summarized in Table 2. Routine dopamine transporter SPECT had been performed, confirming a reduction in striatal dopamine transporter density in all PD patients. All patients received anti-parkinsonian medication. Three patients were only treated with levodopa/decarboxylase inhibitor. In nine patients, levodopa therapy was combined with a dopamine agonist (*n* = 8) or COMT inhibitor (*n* = 1). The remaining three patients were treated with a dopamine agonist and MAO-B inhibitor (*n* = 1), dopamine agonist, levodopa and MAO-B-inhibitor, (*n* = 1) or dopamine agonist, levodopa and COMT inhibitor (*n* = 1). Levodopa equivalent daily dose (LEDD) was calculated based on patients' anti-parkinsonian medication using the conversion factors suggested by Tomlinson et al. (25). Nine PD patients and one healthy control participant received prescribed laxatives daily (Macrogol, Sodium Picosulfate), which was continued throughout the study.

**Table 2:** Demographic and clinical data of patients with Parkinson's disease (PD) and healthy controls.

	**Healthy controls**	**PD patients**	***P*-value**
Sex (male/female)	11/4	9/6	
Age (years)	60.5 ± 9.4	64.6 ± 5.6	0.16
Body mass index (kg/m^2^)	25.8 ± 3.2	25.7 ± 3.6	0.96
Levodopa equivalent dose (mg)	–	599 ± 256	
Disease duration (years)	–	4 (2–6)	
NMS-Questionnaire (NMS-Quest)^*^			
Total NMS-Quest score	0 (0–1)	8 (4–15)	<0.0001
**Scintigraphic measurement of gastric emptying**			
AUC (% x hour)	123 (91–151)	120 (82–161)	0.68
Blood flow (BF) measurement in SMA^*^
Postprandial increase in BF (l/min)	0.74 (0.49–0.95)	0.43 (0.23–0.69)	0.006
Time to maximum (min)	28.9 (22.7–37.9)	30.3 (20.5–51.1)	0.68
Blood glucose (BG) measurement^*^			
Postprandial BG increase (mmol/l)	2.2 (1.8–3.5)	1.95 (1.3–3.4)	0.08
Time to maximum (min)	58.5 (43.1–63.7)	49.4 (38.6–63.3)	0.33

*Data given as mean ± SD or median (10% quantile**–**90% quantile) as appropriate*.

“*” *marks preceding measurements based on the subjects (n = 14 PD patients and 12 healthy controls), who participated in serial MRI-based measurements of blood flow in the superior mesenteric artery (SMA) interrupted by interspersed blood glucose measurements (23), and subsequently participated in the present study between 2 weeks and up to maximal 7 months after. NMS, Non-Motor Symptoms; AUC, Area under the gastric emptying curve*.

### Scintigraphic Measurements

Scintigraphic measurements were performed with PD patients in an “ON-medication” state to avoid motor discomfort and to secure comparability with previous PC-MRI measurements. Participants were instructed to fast for solids and liquids for at least 8 hours prior to scintigraphy. After arrival and a couple of minutes of rest participants ingested a standardized meal consisting of 80 g bread, 30 g jam and 120 g omelet, which was radiolabeled with 40 MBq ^99m^Tc human albumin colloidal particles (Nanocoll), and 120 ml water (26). The participants were given 10 minutes to intake the standardized meal before the first scintigraphic imaging.

**Figure 1:**
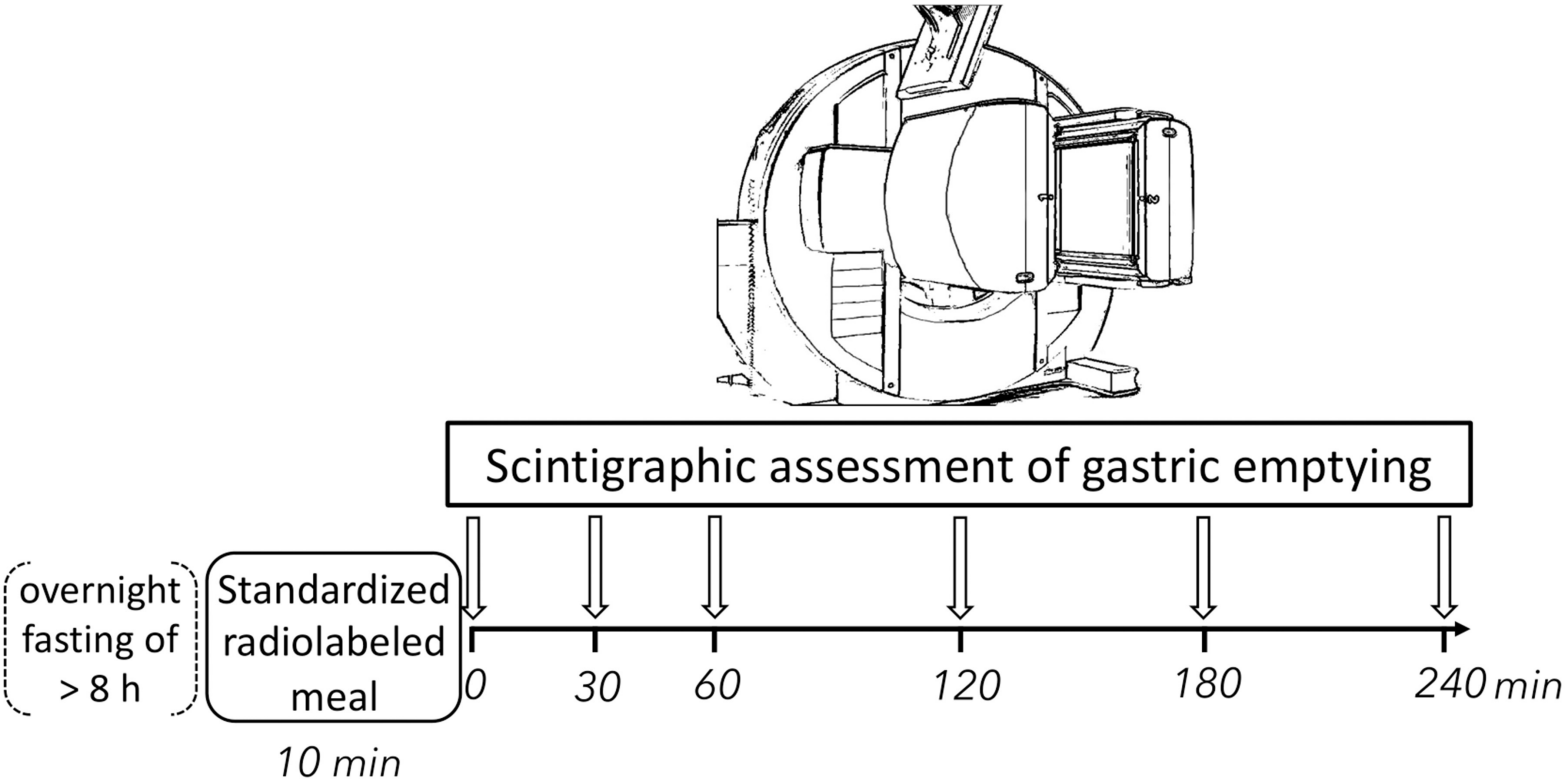
Experimental procedures. Participants ingested a standardized radiolabeled test meal consisting of 80 g bread, 30 g jam and 120 g omelet, which was radiolabeled with 40 MBq ^99m^Tc human albumin colloidal particles (Nanocoll), and 120 ml of water. Gastric emptying was assessed using static scintigraphy of the abdominal region for 2 minutes in both anterior and posterior projection in upright position at six timepoints following meal intake. h, hour; min, minutes.

Two-minute images were obtained at 0, 30, 60, 120, 180, and 240 minutes after finishing the standardized radiolabeled test meal in the anterior and posterior projections (Figure 1). Dynamic scintigraphy was carried out with a dual-head gamma camera equipped with low energy, general purpose collimators (Symbia Intevo, Siemens Healthcare GmbH, Erlangen, Germany) at the Department of Clinical Physiology and Nuclear Medicine, Copenhagen University Hospital Amager and Hvidovre. Regions of interest for integration of radioactivity were drawn manually on all images by one of two experienced investigators (JLM and MD). The obtained counts were corrected for time decay. Geometric mean values of anterior and posterior counts were used for attenuation correction. For each participant, the gastric retention percentage values measured at a given time point (*y*-axis) were plotted against time (*x*-axis). The area under the curve (AUC) was calculated using the trapezoidal rule and expressed as retention percent *x* hour (27). In this study the AUC was used as summary metric of gastric emptying. Gastric emptying was incomplete after 240 minutes in three participants.

### Blood Flow and Blood Glucose Measurements

Blood flow and blood glucose measurements were based on data from a subgroup of 14 PD patients and 12 healthy control subjects, which have been published recently in a larger cohort (23). These subjects participated in serial phase-contrast MRI-based measurements of blood flow in the superior mesenteric artery interrupted by interspersed blood glucose measurements in response to a standardized test meal (~400 kcal) intake, and subsequently participated in the present scintigraphic study. The time elapsed between PC-MRI measurements and scintigraphy ranged between 2 weeks and 7 months. For a detailed description of blood flow and blood glucose measurements, we refer to Siebner et al. (23). We tested for between-group differences in magnitude and time to maximum of the postprandial increase in SMA blood flow and glucose levels.

### NMS-Quest

Participants completed the Non-Motor Symptoms Questionnaire (NMS-Quest) describing subjective non-motor symptoms on arrival at the day of MRI measurements. The NMS-Quest consists of 30 items grouped into nine domains (28, 29). The score ranges from minimum 0, indicating absence of non-motor symptoms, to maximum 30, indicating presence of all non-motor symptoms in question. Recall period was the month before participation. The four study subjects, who did not participate in MRI measurements, completed the NMS-Quest on arrival at the day of scintigraphic measurements.

### Statistical Analysis

Statistical analysis was performed using R software (RStudio Inc., Version 1.2.1335). Normality of the demographic and clinical group data was tested using Shapiro-Wilk test reported as mean ± standard deviation or median and 10%- & 90%-quantiles as appropriate in Table 2. Unpaired two-samples *t*-test was used to test for differences between groups in case of normal distribution. Between-group differences were analyzed using Wilcoxon rank-sum test when normal distribution could not be assumed. Statistical significance was accepted at *p* < 0.05.

To assess whether the postprandial blood flow was associated with gastric emptying, we tested for a relationship between the magnitude and time to maximum of the postprandial increase in SMA blood flow and gastric emptying calculated as AUC using Spearman's rank correlation (*p* < 0.025, Bonferroni corrected for multiple comparisons). We performed additional correlational analyses for exploratory purposes, testing for relationships between individual gastric emptying and age, blood glucose measurements, NMS-Quest score, disease duration and LEDD, using Spearman's rank correlation and applying an adjusted significance level of *p* < 0.0083 as statistical threshold after Bonferroni correction.

## Results

All participants completed the experimental procedures without any adverse events. Demographical and clinical data are presented in Table 2. All patients with PD had bilateral or midline involvement with or without impairment of balance, but were physically independent, corresponding to Hoehn and Yahr stages 2 and 3 (30). Patients had consistently higher NMS-Quest scores than healthy controls (*p* < 0.0001, Table 2). No differences in age, sex or body mass index were found between the two groups (Table 2).

### Scintigraphic Measurements of Postprandial Gastric Emptying

**Figure 2:**
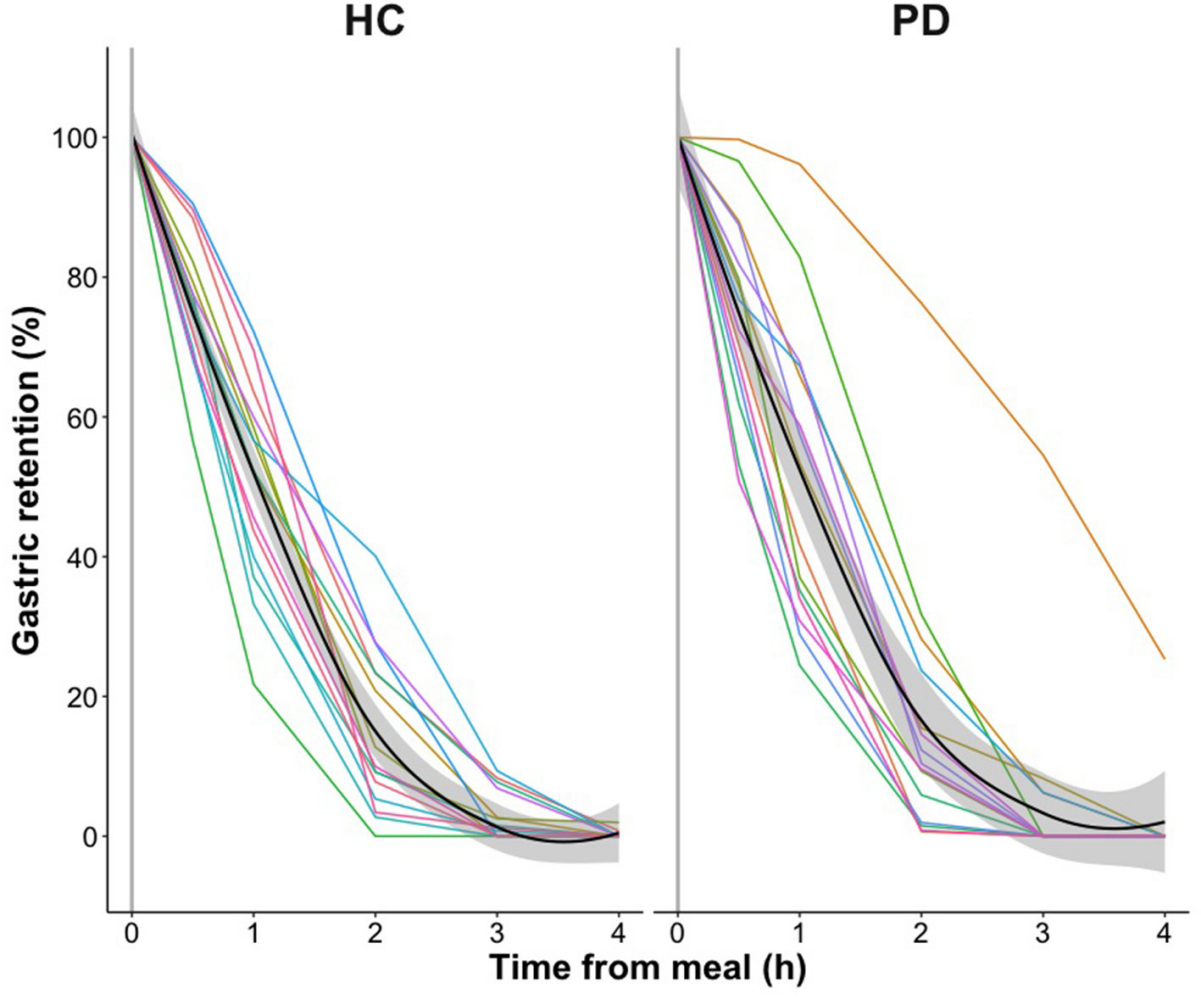
Gastric emptying in healthy control participants (HC) and patients with Parkinson's disease (PD). Individual gastric emptying of the radiolabeled standardized test meal shown as gastric retention at *t* = 0, 30, 60, 120, 180, and 240 min after meal intake with measurements connected using linear interpolation. Mean gastric retention and standard error of the two groups are visualized by fitting a Local Polynomial Regression (LOESS) function: Mean values, black curves; standard error, shaded areas. (h), hour.

**Figure 3:**
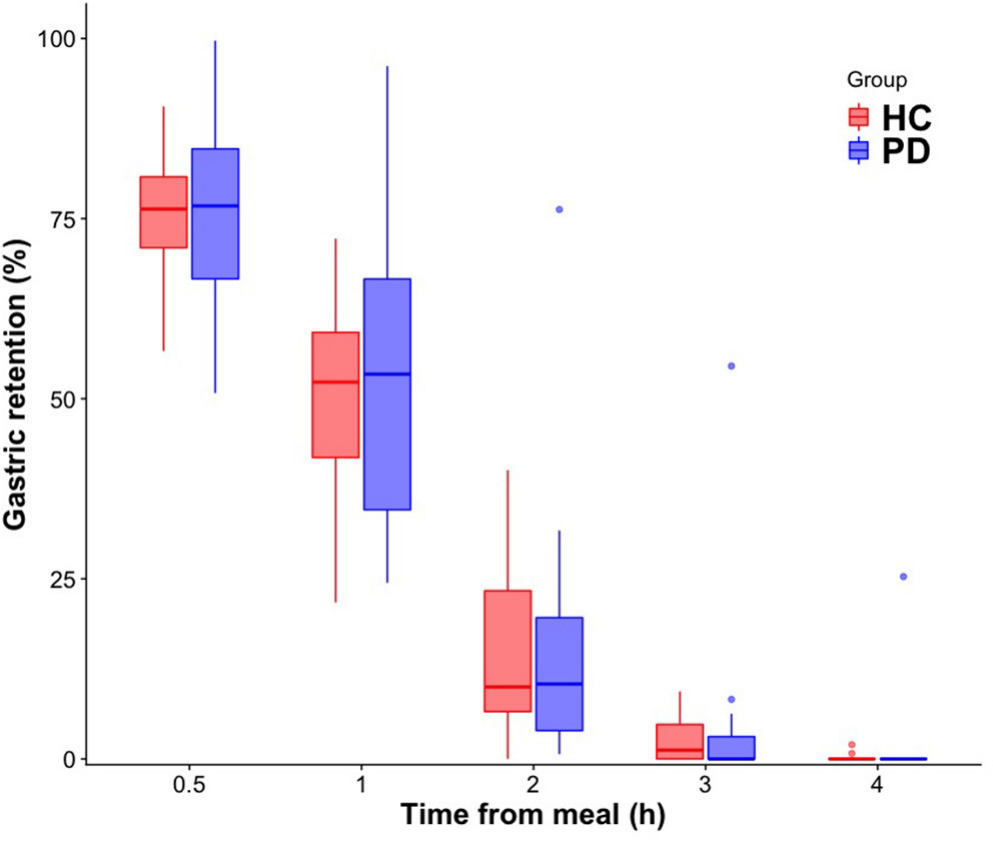
Boxplot of gastric emptying measurements in healthy controls (HC) and patients with Parkinson's disease (PD). Boxes represent the interquartile range with horizontal lines indicating the median of the gastric retention in each group at *t* = 0, 30, 60, 120, 180, and 240 min. Whiskers represent the smallest and the largest values within 1.5 times the 25 and 75th percentiles, respectively. Dots represent extreme outlier values (i.e., values above 1.5 times the interquartile range). (h), hour.

At the group level, gastric emptying calculated as AUC was not different between patients and healthy controls (*p* = 0.68, Table 2). The dynamics of gastric retention at the individual and group level are presented separately for PD patients and healthy participants in Figure 2, showing substantial inter-subject variability in both groups. While the individual slopes of gastric retention over time varied considerably, the distribution of gastric retention over time was very similar between groups, resulting in almost identical AUC in PD patients and healthy controls (Table 2). The only exception was a single PD patient who showed a markedly increased gastric retention at all measurements and met the definition of delayed gastric emptying (26). This patient responded well to antiparkinsonian drug treatment, had a 5-year disease duration, was successfully treated with Macrogol for constipation and reported no upper gastrointestinal dysfunction symptoms, why we had no reason to question the diagnosis of probable PD. Apart from this patient, all participants showed <2% gastric retention 4 h after meal intake. A boxplot presentation of the individual gastric retention in PD patients and healthy controls shows the same pattern, confirming a very similar distribution of postprandial gastric retention of the ingested meal in the PD and healthy control groups (Figure 3).

### Postprandial Blood Flow and Blood Glucose Changes

In the 14 PD patients and 12 healthy subjects who had participated in PC-MRI prior to scintigraphic measurements, the absolute postprandial increase in SMA blood flow was attenuated in PD compared to healthy controls (*p* = 0.006, Table 2). A statistical trend toward an attenuated postprandial increase in blood glucose level was also observed in the patient group compared to the healthy control group (*p* = 0.08, Table 2). There was no significant between-group difference in time to reach maximal blood flow and time to reach maximum blood glucose level. In summary, the main findings of our previous study in a larger cohort (23) were still present in this subsample.

### Correlation Analysis

Our correlation analysis revealed no significant relationships between gastric emptying as indexed by AUC and the magnitude or the time to maximum of the postprandial increase in SMA blood flow (Table 3). Exploratory analyses showed a trend toward significant positive correlation between gastric emptying and NMS-Quest score in PD patients (rho = 0.57, *p* = 0.03) based on Bonferroni correction. A longer gastric emptying was associated with a higher subjective prevalence of non-motor symptoms. Hence, the longer the individual gastric emptying, the higher the individual NMS-Quest score. Also, exploratory analyses showed that individual gastric emptying did not correlate with age, blood glucose measurements, disease duration or LEDD (Table 3).

**Table 3:** Correlations of clinical data patients with Parkinson's disease (PD) and healthy controls.

**Variables**	**All subjects**		**Healthy controls**		**PD patients**	
	**Rho**	***p*-value**	**Rho**	***p*-value**	**Rho**	***p*-value**
AUC vs. Blood flow increase	−0.113	0.58	−0.074	0.82	−0.328	0.25
AUC vs. Blood flow time to maximum	0.049	0.81	−0.161	0.62	0.253	0.38
**Exploratory analyses**						
AUC vs. Age	−0.12	0.53	0.106	0.71	−0.217	0.44
AUC vs. Blood glucose increase	−0.216	0.29	−0.085	0.79	−0.232	0.43
AUC vs. Blood glucose time to maximum	−0.248	0.22	−0.613	0.03^*^	−0.064	0.83
AUC vs. NMS-Quest score	0.17	0.37	0.338	0.22	0.57	0.03^*^
AUC vs. Disease duration					−0.104	0.71
AUC vs. LEDD					0.229	0.41

*Correlations are expressed as Spearman's rank correlation coefficient (ρ = rho). Gastric emptying is expressed as area under the curve (AUC). LEDD, Levodopa equivalent daily dose; NMS-Quest, Non-Motor Symptoms Questionnaire*.

Significant p-values are corrected for multiple comparisons based on Bonferroni correction. For primary variables an adjusted significance level of p < 0.0025 is applied, while for exploratory analyses significant p-values are adjusted to p < 0.0083 as the statistical threshold. Based on Bonferroni correction there is no significant p-value, while trend toward significance is expressed by “^*^”.

## Discussion

In a small cohort of early-stage PD patients who continued their usual dopamine replacement therapy, we found no delay in gastric emptying after a solid meal challenge in 14 of 15 patients compared to age- and sex-matched healthy controls with comparable body mass index. Only one of 15 patients included in this study showed delayed gastric emptying. Most patients had participated in our recent PC-MRI study and had shown an attenuated postprandial increase in SMA blood flow. In these patients, gastric emptying did not correlate with the magnitude of the postprandial blood flow increase in the SMA nor with the time to reach the postprandial peak of SMA blood flow.

### Gastric Emptying

When studying gastric function in PD, the influence of the age of participants, disease severity and medication state needs to be considered. Although the available literature is contradictory regarding the effect of healthy aging on gastric emptying, recent studies support age-related slowing of gastric emptying (31, 32). The impact of aging on gastric emptying may also apply to PD patients. A previous scintigraphy study reported a prolongation of gastric emptying in PD patients in an “ON-medication” state, both with response fluctuations after long-term levodopa therapy and without response fluctuations, compared to a healthy control group (13). Since healthy control participants were on average more than 15 years younger than the patients with PD, an effect of age on gastric emptying may account for the between-group difference. Another study using an electromagnetic capsule system showed that gastric transit time was longer in patients with PD compared with healthy subjects who were on average more than 40 years younger (22). The authors suggested that the longer gastric transit time could be due to three outliers but did not exclude an age-related decrease in gastric motility. In fact, a previous study using a similar electromagnetic capsule system showed no difference in gastric transit time in PD compared to age- and sex-matched controls (33), pointing to a relevant influence of age on gastric emptying. Future studies need to systematically examine how healthy aging impacts gastric emptying and how aging influences gastric emptying in PD. In our cross-sectional study, gastric emptying did not correlate with age (Table 3), but the relatively narrow age range of our study population does not allow us to dismiss an effect of aging on gastric emptying. Taken together, there is prevailing evidence against a prolongation of gastric emptying in patients with PD in the “ON-medication” state, when patients are compared to age-matched groups of healthy individuals.

Only one previous scintigraphy study has investigated the influence of disease severity on gastric emptying in PD patients. Hardoff et al. compared gastric emptying between patients with mild PD (Hoehn and Yahr stage 1–2) and patients with moderate PD (Hoehn and Yahr stage 2.5–3.0) and found no significant difference, however including PD patients in different medication states (14). In our study, we included PD patients with Hoehn and Yahr stages 2 and 3 in relatively early stages of the disease (mean disease duration of 3.9 ± 2.2 years; LEDD of 599 ± 256 mg) and only one patient had delayed gastric emptying. As mentioned, we had no reason to question this patient's diagnosis of probable PD. Neither Hardoff et al. nor our study did examine gastric emptying in later disease stages of PD. We can therefore not exclude that impaired gastric emptying might be present in a smaller subgroup of PD patients in the first years after the emergence of parkinsonism. Delayed gastric emptying may be more prominent in later disease stages and needs to be investigated in future studies.

Erratic delivery of levodopa from the stomach to its duodenal and jejunal absorption sites due to delayed gastric emptying has been suggested to be a causative factor for inconsistent response to levodopa and consequently motor fluctuations in PD (1–4, 9, 10). PD patients with response fluctuations after long-term levodopa therapy showed delayed GET compared to non-fluctuating patients (13). Another study found a significant relationship between levodopa pharmacokinetics and gastric emptying and could show that delayed gastric emptying was more common in PD patients with a late plasma levodopa peak (34). In addition, motor fluctuations and dyskinesia in PD patients were considerably improved by continuous jejunal levodopa infusion and thereby bypassing gastric emptying (35). Interestingly, subthalamic deep brain stimulation seems to improve gastric emptying (36, 37). Therefore, the emergence of dysfunctional gastric emptying during disease progression warrants further studies.

We studied PD patients under therapy to secure comparability with previous PC-MRI measurements. Furthermore, we expected the PD patients to be stressed and having more difficulties to cooperate during the MRI session, if they had been in an “OFF-medication” state. Therefore, it remains unclear whether dopaminergic medication affects gastric emptying or vice versa. Several studies showed that a single oral dose of levodopa inhibits gastric emptying in both young and elderly healthy subjects (38–40). However, this might not to be the case in patients with PD, as no difference in breath test GET was found in PD patients before and approximately 1.5 years after the initiation of levodopa treatment (41). Another scintigraphy study observed no significant difference in GET between levodopa-treated and untreated PD patients, and the same was evident when dividing the patient group in mild and moderate PD (14). Lastly, a scintigraphy study showed that PD patients in a defined “OFF-medication” state (withdrawn from anti-parkinsonian medication for 12 h) had significantly faster mean gastric emptying than controls (16). Taken together, there are apparent discrepancies in the reported findings with some studies pointing to an interaction between gastric emptying and levodopa. Even though our current findings are in line with the most recent study, which observed no delayed GET in PD patients in the “ON-medication” state (18), the interaction between levodopa treatment and gastric emptying is still not fully understood and needs further investigation.

Gastric emptying can also be indirectly tested using the ^13^C-octanoate breath test, which measures the concentration of ^13^CO_2_ expired in the lungs after intake of a meal containing ^13^C-sodium-octanoate. In contrast to the scintigraphy studies described above, most studies employing the ^13^C-octanoate breath test reported abnormal results in PD patients (11). Since ^13^C-sodium-octanoate is absorbed in the jejunum and metabolized in the liver to ^13^CO_2_, the ^13^C-octanoate breath test is not only dependent on mechanical stomach emptying, but also on small intestinal motility, ^13^C-octanoate absorption and hepatic metabolism (11, 42). It is worth noting that small intestinal absorption has been shown to be deranged and small intestinal transit time to be increased in patients with PD (33, 43). Hence, abnormal small intestine absorption and motility rather than delayed gastric emptying may account for the “abnormal” ^13^C-octanoate breath test findings and normal GET as revealed by the most recent gastric scintigraphy study (18).

*Post-hoc* exploratory analysis revealed that gastric emptying tended to scale positively with the prevalence of non-motor symptoms in the group of PD patients. This might suggest that PD patients with higher subjective prevalence of non-motor symptoms might be in greater risk of suffering delayed gastric emptying than others. However, the NMS-Quest does not include upper gastrointestinal symptoms apart from dysphagia, and gastric emptying was on average not different from healthy controls. Future studies need to validate whether gastric emptying may be useful as a marker of overall non-motor involvement.

### Relation Between Gastric Emptying and Postprandial SMA Blood Flow

This study is a follow-up examination and was prompted by our recent postprandial phase-contrast MRI study (23). Since 14 out of 15 patients and 12 out of 15 healthy controls had been taken part in serial PC-MRI measurements of postprandial blood flow in the SMA, we were able to correlate the gastric emptying measurements with the blood flow measurements of the SMA. Even though we were only able to reexamine a subgroup of the original cohort, we still found the significant attenuation of postprandial increase in SMA blood flow and a trend toward a significant attenuation of postprandial increase in blood glucose level in this reduced sample size of the original cohort. The time from food intake to the postprandial peak in SMA blood flow was not different between groups. We have previously discussed in detail the possible mechanisms behind the impaired functional regulation of postprandial gastrointestinal perfusion in PD (23). We had considered that changes in motility, impaired glucose absorption as well as impaired neural control of gastrointestinal perfusion may be affected in PD and thereby explain the attenuated postprandial response in both blood flow and glucose. This consideration prompted this follow-up examination. Our aim was to correlate the gastric emptying measurements with the previous blood flow measurements of the SMA and thus, to clarify whether changes in gastric emptying could explain the attenuation of the postprandial SMA response. In this sub-cohort, gastric emptying did not correlate with the postprandial blood flow response in the SMA. Gastric emptying correlated neither with postprandial absolute blood flow increase nor with time to reach maximal blood flow in SMA. Albeit our sample size was limited, we tentatively conclude that the attenuated postprandial blood flow in SMA previously seen in PD patients is not related to individual alterations in gastric emptying. In this context, it is important to note, that the SMA supplies the gastrointestinal tract from lower part of the duodenum to left colic flexure (44), but we did not examine small intestinal or colonic gastrointestinal motility in this study. We can therefore not exclude that gastric emptying in PD may be related to changes in postprandial blood flow response in the arteries supplying the stomach. When piloting the PC-MRI study, it was not possible to establish reliable blood flow measurements in the celiac artery or left gastric artery supplying the stomach, spleen, and liver (44). This precluded correlation analyses between postprandial blood flow dynamics from the arteries suppling the stomach with gastric emptying in the current study. Future studies need to measure postprandial blood flow in both the celiac artery with its branches and SMA, and concurrently examine the temporal dynamics of gastric, small intestinal and colonic motility in order to correlate blood flow in the supplying arteries with the temporal dynamics of the respective sections of the gastrointestinal tract.

Given the small sample size and methodological differences in the meal test (e.g., type of meal, body position during measurement), the overall clinical significance of impaired functional regulation of postprandial mesenteric blood flow response in PD remains still to be clarified. Future studies need to clarify whether an attenuated postprandial SMA blood flow response reflects gastrointestinal dysfunction. Longitudinal study designs will be needed to link changes in postprandial SMA blood flow to the course of disease, especially whether it can be useful as progression marker in PD or in prodromal PD.

In this study, gastric emptying function was not affected in a group of PD patients, although gastrointestinal symptoms are frequently reported in PD (1–4). Symptoms may thus be related to impaired function of the middle and lower portion of the gastrointestinal tract. In fact, previous studies showed significantly delayed small intestinal and colonic transit times in PD patients compared to healthy control subjects (18, 33). The impaired gastrointestinal motility in the small intestine and colon could be a contributory mechanism to the attenuated postprandial blood flow in SMA and glucose response seen in PD patients in our study. Also, the abnormal small intestine motility in PD could together with the deranged absorption explain why most ^13^C-octanoate breath tests show delayed gastric emptying in PD in conflict with studies employing gastric scintigraphy (42, 43). Motility impairment of the lower gastrointestinal tract might more be decisive for subjective symptoms. Conversely, the attenuated postprandial blood flow response in SMA may not contribute substantially to clinical gastrointestinal symptoms but might be an independent marker for gastrointestinal involvement in PD.

## Limitations

The current study has several limitations. The number of patients and healthy controls included in this study is relatively small. Because of the limited statistical power, we cannot exclude subtle between-group differences in gastric emptying between patients with PD and healthy controls. However, group sizes were comparable with the sample sizes of previous scintigraphy studies (Table 1), and we were able to retrieve the between-group difference in post-prandial blood flow increase in this subsample of our recent published PC-MRI study. The range of gastric emptying was very similar in both groups and both groups showed substantial inter-individual variations in gastric emptying. Therefore, we argue that our preliminary results speak against a substantial prolongation in gastric emptying in PD with a relatively short disease duration.

The PC-MRI measurements of blood flow were performed in a supine position and the scintigraphic measurements of gastric emptying were performed in a standing position. PC-MRI measurements preceded scintigraphic measurements and the time elapsed between both measurements varied from weeks to months. Also, the content of the meal challenges differed between the PC-MRI and scintigraphic measurements. These methodological differences may have obscured a weak correlation between SMA blood flow response and gastric emptying.

The sequential nature of our follow-up examination is a methodological limitation. When planning the MRI study, we initially considered to interleave measurements of gastric emptying with PC-MRI measurements of SMA blood flow. This would have provided concurrent measurements of the response to a single standardized test meal under comparable conditions. Also, participants could have been spared a radiation dose by using MRI rather than scintigraphy to assess gastric emptying. We opted against interleaved MRI measurements of SMA blood flow and gastric emptying because of practical considerations. Different acquisition techniques are used for assessing SMA blood flow and gastric emptying. This would have greatly compromised the temporal resolution of each measurement. Adding measurements of gastric emptying would have been at the expense of blood flow measurements, thereby give a less accurate picture of the blood flow response. The present study may inform the optimal timing of future interleaved MRI measurements by revealing the temporal postprandial dynamics of both, SMA blood flow and gastric emptying.

We did not perform a detailed assessment of motor impairment at the day of examination (e.g., Unified Parkinson's Disease Rating Scale). Instead, we used disease duration and LEDD as proxies for disease severity. Therefore, our study provides no clues whether overall motor dysfunction is positively correlated with gastric emptying.

## Conclusions

Our results obtained in 15 patients with early-stage PD suggest that variations in gastric emptying after solid meal intake is within the normal range in the majority of cases in the “ON-medication” state. There is also no evidence for a tight relationship between the attenuated postprandial blood flow response in the SMA and normal variations in gastric emptying. These findings are preliminary and need to be corroborated in larger cohorts to enable generalization of our findings. It also remains to be addressed if gastric emptying is delayed in later disease stages.

## Data Availability Statement

The datasets presented in this article are not readily available because the dataset is only pseudonymized and cannot be shared without a formal Data Processing Agreement or a formal approval by the Danish Data Protection Agency in order to meet the requirements of the GDPR and to ensure the protection of the rights of the data subject. Requests to access the datasets should be directed to Thomas H. Siebner, thomas.hartwig.siebner@regionh.dk.

## Ethics Statement

This study involving human participants was reviewed and approved by Regional Committee on Health Research Ethics of the Capital Region of Denmark (H-18054923). The patients/participants provided their written informed consent to participate in this study.

## Author Contributions

HS, JM, JH, AL, FB, and TS: research project—conception. TS and JM: research project—organization. JM, MD, and TS: research project—execution. SF, TS, CM, and HS: statistical analysis—design. SF and TS: statistical analysis—execution. HS, CM, JM, MD, FB, AL, and JH: statistical analysis—review and critique. TS: manuscript—writing of the first draft. HS, JM, MD, CM, FB, AL, SF, and JH: manuscript—review and critique. All authors contributed to the article and approved the submitted version.

## Funding

This study did not receive any extramural funding and was exclusively financed by the Danish Research Centre for Magnetic Resonance. HS was supported by a Lundbeck Foundation collaborative project ADAptive and Precise Targeting of cortex-basal ganglia circuits in Parkinson's Disease (Grant No. R336-2020-1035) and holds a 5-year professorship in precision medicine at the Faculty of Health Sciences and Medicine, University of Copenhagen which is sponsored by the Lundbeck Foundation (Grant No. R186-2015-2138). CM is completing a PhD, which is sponsored by the Independent Research Fund Denmark (Grant No. 7016-00226B) and by a grant from the Parkinsonforeningen (Danish Parkinson Association) (Grant No. A289).

## Conflict of Interest

HS has received honoraria as speaker from Sanofi Genzyme, Denmark and Novartis, Denmark, as consultant from Sanofi Genzyme, Denmark and as editor-in-chief (Neuroimage Clinical) and senior editor (NeuroImage) from Elsevier Publishers, Amsterdam, the Netherlands and has received royalties as book editor from Springer Publishers, Stuttgart, Germany and from Gyldendal Publishers, Copenhagen, Denmark. FB has received honoraria as a consultant for Ferring Pharmaceuticals, Denmark. AL has received honoraria as speaker from AbbVie, United States. The remaining authors declare that the research was conducted in the absence of any commercial or financial relationships that could be construed as a potential conflict of interest.

## Publisher's Note

All claims expressed in this article are solely those of the authors and do not necessarily represent those of their affiliated organizations, or those of the publisher, the editors and the reviewers. Any product that may be evaluated in this article, or claim that may be made by its manufacturer, is not guaranteed or endorsed by the publisher.
